# Hybrid Materials—Mg_3_Al-LDH/Ionic Liquids/Chitosan Used in the Recovery Process of Pd Ions from Aqueous Solutions

**DOI:** 10.3390/molecules29246001

**Published:** 2024-12-19

**Authors:** Emilia Milos, Laura Cocheci, Adriana Popa, Lavinia Lupa, Anca Filimon

**Affiliations:** 1 Faculty of Chemical Engineering, Biotechnologies and Environmental Protection, Politehnica University Timisoara, Victoriei Square, No. 2, 300006 Timisoara, Romania; emilia.milos@student.upt.ro (E.M.); laura.cocheci@upt.ro (L.C.); 2 “Coriolan Drăgulescu” Institute of Chemistry, Bv. Mihai Viteazul, No. 24, 300223 Timisoara, Romania; apopa@acad-icht.tm.edu.ro; 3 “Petru Poni” Institute of Macromolecular Chemistry, Grigore Ghica Alley 41A, 700487 Iasi, Romania; afilimon@icmpp.ro

**Keywords:** layered double hydroxides, chitosan, ionic liquids, palladium, recovery

## Abstract

The recovery of palladium from aqueous solutions is important due to its critical role in various industrial applications and the growing demand for sustainable resource management. This study investigates the potential of hybrid materials composed of Mg_3_Al layered double hydroxides (LDHs), chitosan, and ionic liquids (methyl trialchil ammonium chloride) for the efficient adsorption of palladium ions from low-concentration aqueous solutions. Comprehensive characterization techniques, including X-ray diffraction (RX), Fourier transform infrared spectroscopy (FTIR), scanning electron microscopy (SEM), energy-dispersive X-ray spectroscopy (EDX), and thermogravimetric analysis (TG), were employed to elucidate the structural and compositional properties of the hybrid materials. The results of the batch adsorption experiments demonstrate that each component contributes synergistically to the adsorption process, significantly enhancing the overall efficacy of palladium recovery. Furthermore, the method of preparing the adsorbent material was found to impact the effectiveness of palladium recovery. Among the materials tested, the chitosan/Mg_3_Al/IL hybrid exhibited the highest adsorption capacity (q_max_ = 98 mg/g), suggesting that the ionic liquid functionalization is most beneficial when applied during the hybrid material synthesis, rather than during the LDH synthesis process. This research underscores the viability of hybrid materials as a sustainable approach to palladium recovery, contributing to advancements in environmental remediation technologies.

## 1. Introduction

Palladium, a rare, silver-white metal discovered in 1803 by William Hyde Wollaston, is part of the platinum group metals (PGMs) along with platinum, rhodium, ruthenium, iridium, and osmium. It is highly valued for its ductility, malleability, and various industrial applications. Annually, around 300 tons of palladium are produced, primarily in Russia, South Africa, and North America. The majority of palladium is used in the automotive industry for catalytic converters, which help control emissions from exhaust gases. Additionally, palladium is found in electronic devices, jewelry, and dental applications [1,2,3,4]. Due to the depletion of primary reserves and the fluctuating price of palladium, it is essential to recover palladium from secondary products and waste, as it significantly impacts both environmental sustainability and the economy. As environmental regulations become stricter, recycling palladium waste reduces dependence on mining, which is often ecologically harmful. This practice not only optimizes extraction costs but also promotes technological innovations in recovery processes, contributing to the development of more efficient and eco-friendly methods. Additionally, research in this field can influence resource management policies, highlighting the importance of a circular economy that values available resources [3,5,6]. In addition to solid waste containing palladium generated from industrial activities and various recycling processes, substantial quantities of aqueous solutions with palladium content are also produced. The recovery of palladium from aqueous solutions can be accomplished using a variety of methods, each offering distinct advantages and limitations. Palladium has been successfully recovered from acidic leachate solutions through precipitation methods. Although this approach demonstrates high efficiency, it necessitates the use of expensive and potentially hazardous reagents, as well as meticulous control of pH and reagent dosage. Additionally, this method may not be effective for the removal of trace concentrations of palladium ions [7,8,9,10]. Electrochemical recovery is another technique where an electric current is applied to precipitate palladium onto an electrode, allowing for selective recovery. This method can be highly efficient but may involve higher operational costs and complex equipment [11,12,13]. Additionally, membrane separation techniques, such as nanofiltration or ultrafiltration, can be utilized to separate palladium from other ions based on size or charge. These methods require significant energy input and maintenance and developed a yield of palladium recovery of 66% [14]. Lastly, ion exchange resins can selectively capture palladium ions from solutions, providing a high degree of specificity. While effective, this method can be limited by the resin’s capacity and the need for regeneration [15]. In addition to the aforementioned techniques, adsorption represents one of the most efficient methods for palladium recovery [16,17]. This process involves the use of various adsorbent materials, which can selectively bind palladium ions from aqueous solutions. Adsorption offers several advantages, including the ability to operate effectively at low concentrations of palladium, a relatively simple operational process, and the potential for regeneration of the adsorbent, which enhances sustainability. Furthermore, advancements in the development of novel adsorbent materials, such as functionalized materials, have significantly improved the efficiency and selectivity of palladium recovery through adsorption, making it a promising option in both industrial and environmental applications [18,19,20,21]. Based on these considerations, this study focuses on using hybrid materials based on layered double hydroxides (LDHs) as adsorbents for palladium recovery. LDHs are two-dimensional materials characterized by high porosity and the presence of exchangeable anionic species. Structurally, they consist of cationic metal layers intercalated with hydrated anions in the interlayer spaces [22]. Due to their large surface area and excellent ion exchange properties, LDHs serve as highly effective adsorbents for wastewater treatment, demonstrating the capacity to capture a wide range of ions and molecules from aqueous solutions [23,24,25]. However, to enhance their adsorptive efficiency, the surface of these materials can be functionalized. Functionalization involves modifying the surface properties of LDHs to improve their interaction with specific target contaminants, such as palladium ions. By introducing functional groups or altering the surface structure, the adsorption capacity and selectivity of LDHs can be significantly increased, making them even more effective for applications in environmental remediation and resource recovery [26,27,28]. For these reasons, in the present paper, we employed ionic liquids for the functionalization of LDHs. Ionic liquids are unique solvents composed entirely of ions, which exhibit distinct properties such as low volatility, high thermal stability, and tunable solubility [29,30]. These characteristics make ionic liquids particularly suitable for modifying the surface of LDHs. The ionic liquid can interact with the LDH surface, facilitating the incorporation of functional moieties that improve binding interactions. This approach not only enhances the efficiency of palladium recovery but also allows for better control over the adsorption process. Additionally, the use of ionic liquids can provide a more environmentally friendly alternative to traditional organic solvents, aligning with sustainable practices in material science. To further enhance the mechanical strength of the obtained hybrid materials, chitosan was also incorporated into the formulation. Chitosan, a biopolymer derived from chitin, is known for its excellent mechanical properties, biodegradability, and non-toxicity [31,32]. By integrating chitosan into the hybrid materials, we aim to improve their structural integrity and durability. The addition of chitosan not only reinforces the mechanical strength of the hybrid materials but also contributes to their stability in aqueous environments. This is particularly important for applications involving adsorption processes, where the materials may be subjected to varying conditions such as agitation or changes in pH. Enhanced mechanical resistance ensures that the adsorbent maintains its structural integrity during use, thereby prolonging its lifespan and effectiveness. Moreover, chitosan may also provide additional functional groups that can enhance the adsorption capabilities of the hybrid materials. This dual benefit of mechanical reinforcement and improved adsorptive properties makes chitosan an advantageous component in the development of hybrid adsorbent materials for environmental applications. Therefore, this study investigates the recovery of palladium ions from aqueous solutions using hybrid materials composed of Mg_3_Al-LDH, ionic liquids, and chitosan. The ionic liquid used is methyl-trialkyl-ammonium chloride. The innovative contributions of this study are threefold: (i) the development of advanced adsorbent materials exhibiting enhanced adsorption capacities, attributed to the synergistic interactions among the components within the hybrid materials; (ii) a reduction in preparation costs achieved through the minimal use of ionic liquids and an optimized manufacturing process that minimizes ionic liquid loss to the aqueous phase; and (iii) the development of adsorbent materials with exceptional properties, facilitating their subsequent application in column adsorption studies of palladium and their potential for the recovery of palladium from solutions containing trace concentrations and their further utilization as photocatalysts.

## 2. Results and Discussion

### 2.1. Structural and Morphological Analysis: Evidence of Component Compatibility and Porous Network Formation

**Figure 1:**
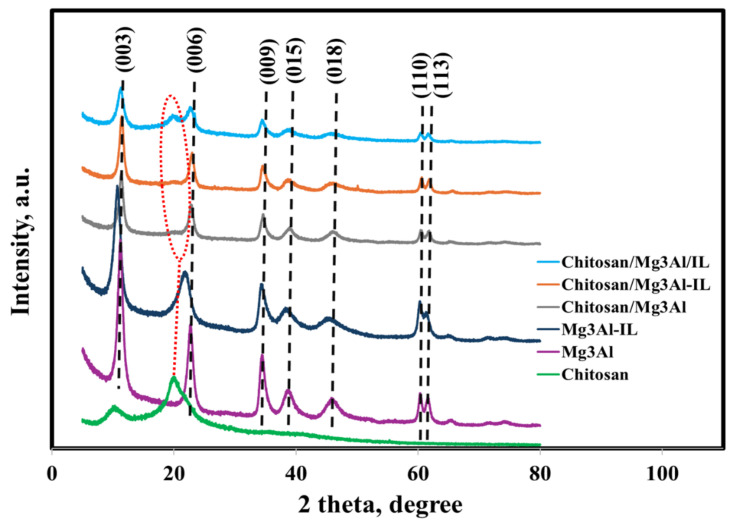
The X-ray diffractograms of the investigated hybrid materials.

The XRD diffractograms of the synthesized hybrid samples are shown in Figure 1 and are compared with the X-ray diffractograms of the raw materials (chitosan, Mg_3_Al, Mg_3_Al-IL).

The X-ray diffractogram reveals that the Mg_3_Al sample, produced through co-precipitation under low supersaturation, has indeed yielded a predominant crystalline phase of layered double hydroxide. It was notable that basal peaks were observed for planes (003) and (006) at angles of 2θ = 11.5° (2θ = 22.8°), as well as the non-basal peaks observed for planes (110) and (113) at an approximate angle of 2θ around 60 degrees. The layered double hydroxide sample, known as Mg_3_Al-IL and functionalized with methyl trialkyl ammonium chloride, shows a noticeable shift in the basal peaks towards smaller 2θ angles. Additionally, there is a reduction in their intensity while the basal spacing increases, suggesting the successful intercalation of the ionic liquid within the layers of LDH. X-ray diffraction analysis of pure chitosan reveals wide peaks observed at angles of 2θ = 10° and 2θ = 20°, findings similar to the data presented in specialized literature [27,29,33,34,35,36]. In the case of hybrid samples, the peak for chitosan at 2θ = 10° is observed to disappear, and the very broad peak at 2θ = 20° has become weak. These results suggest that chitosan has good compatibility with LDH, leading to the formation of porous networks.

**Figure 2:**
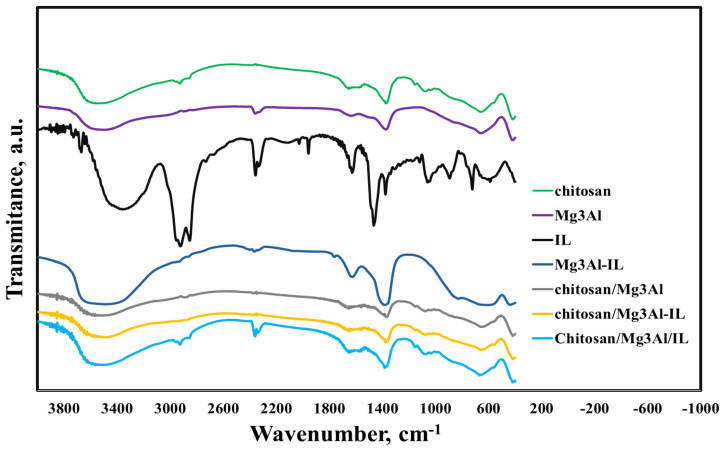
FTIR spectra of the investigated hybrid materials.

In order to analyze the structure and composition of the synthesized hybrid samples, the Fourier transform infrared spectra were analyzed and are presented in Figure 2.

For chitosan, a broad band at 3530 cm^−1^ is observed, which corresponds to the axial stretching of the O-H and N-H bonds, as well as the intramolecular hydrogen bonds. The absorption bands at about 2926 cm^−1^ can be attributed to asymmetric and symmetric C-H stretching. The band at 1570 cm^−1^ corresponds to the N-H bond. Symmetric CH_2_ and CH_3_ deformations were confirmed by the presence of bands at approximately 1423 and 1375 cm^−1^, respectively. The absorption band at 1153 cm^−1^ can be attributed to the asymmetric stretching of the C-O-C bridge. The bands at 1066 and 1028 cm^−1^ correspond to C-O stretching. All bands are found in the spectra of chitosan samples reported by other researchers [26,35,36]. The spectrum of methyl-trialkyl-ammonium chloride ionic liquid shows two absorption bands located at 2920 cm^−1^ and 2852 cm^−1^ attributed to -CH_2_ and -CH_3_ groups, respectively. The quaternary ammonium group was highlighted by the appearance of absorption bands at 1462 cm^−1^ and 1377 cm^−1^ [29]. The FTIR spectrum of Mg_3_Al contains the absorption bands specific to a layered double hydroxide sample with the carbonate anion: the broad band at 3600 cm^−1^ corresponds to O-H stretching vibration of the surface and interlayer water molecules, the band with maxima at 1640 cm^−1^ corresponds to the water bending mode, the bands located at 1370 and 658 cm^−1^ could be assigned to asymmetric stretching vibrations and the ν_4_ mode of interlayer CO_3_^2−^ anions, respectively, while the band with maxima at 412 cm^−1^ corresponds to entire octahedral network vibrations [34]. The FTIR spectrum of Mg_3_Al-IL presents, besides all absorption bands characteristic for layered double hydroxide, a shoulder located at 2930 cm^−1^ that can be assigned to the -CH_2_ group in ionic liquid. The absorption band assigned to the water bending mode is shifted to 1620 cm^−1^ compared to 1640 cm^−1^ (Mg_3_Al) confirming the presence of ionic liquid in the structure of Mg_3_Al-IL sample. The spectrum of chitosan/Mg_3_Al shows two overlapping absorption bands in the range of 1250–2500 cm^−1^, resulting in two shoulders located at 1375 and 1365 cm^−1^ instead of a single band with the maximum at 1370 cm^−1^ assigned to asymmetric stretching vibrations of interlayer CO_3_^2−^ anions in the spectrum of Mg_3_Al. The appearance of a band at 1076 cm^−1^ assigned to C-O stretching and a shoulder at 833 cm^−1^ attributed to out-of-plane deformation vibrations (ν_2_) of CO_3_^2−^ confirms that there are interactions between chitosan and layered double hydroxide. The FTIR spectrum of chitosan/Mg_3_Al-IL sample presents, besides specific absorption bands of layered double hydroxide, an absorption shoulder located at 2925 cm^−1^ from ionic liquid and a split band in the range of 1250–2500 cm^−1^ due to chitosan. In addition, the presence of a band situated at 1070 cm^−1^ is observed confirming the interaction between chitosan and layered double hydroxide. Compared to chitosan/Mg_3_Al-IL, with the sample of chitosan/Mg_3_Al/IL being synthesized differently, it has a more evident absorption band specific to the ionic liquid, located at 2925 cm^−1^, and a split band with maxima at 1385 cm^−1^ and shoulder at 1370 cm^−1^ due to chitosan interaction with layered double hydroxide. In the FTIR spectra of the hybrid materials, both the characteristic bands of chitosan and LDH, respectively, were observed, demonstrating that the preparation method was effective for obtaining the studied hybrid materials.

**Figure 3:**
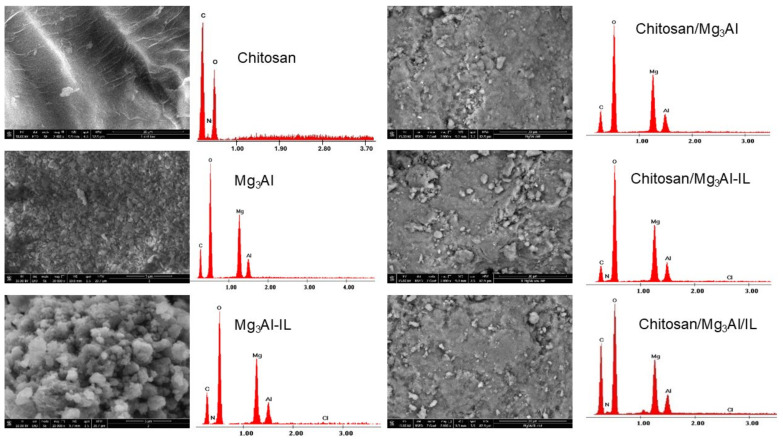
SEM images and EDX spectra of the investigated hybrid materials.

Scanning electron microscopy was used to investigate the surface morphology of the synthesized hybrid materials. SEM images together with EDX spectra are presented in Figure 3. The chitosan particles exhibit a smooth and uniform surface and the Mg_3_Al sample showcases a hexagonal morphology, organized in overlapping layers as is customary for layered double hydroxides. Upon functionalizing it with methyl trialkyl ammonium chloride through co-synthesis, it is observed that the resulting sample displays a network modification. The layers become disordered because of the ionic liquid permeating between them, giving the surface of the sample that can be associated with cotton flowers. The hybrid samples that were synthesized exhibited varying surface morphologies, showcasing notably rough surfaces with small particles that were uniformly dispersed. The EDX spectra provide compelling evidence for the successful design of hybrid materials. This validation is achieved by observing the distinct peaks corresponding to the specific elements of each component that constitutes the hybrid material. These identifiable peaks indicate the presence and proportion of the various elements, confirming that the intended components have been effectively integrated into the hybrid structure. Thus, the EDX analysis not only supports the synthesis process but also underscores the successful formation of the desired hybrid materials.

### 2.2. Thermal Behavior and Stability Analysis

**Figure 4:**
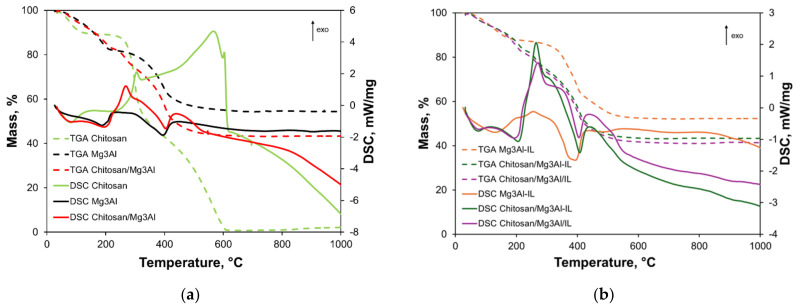
Thermoanalytical curves of: (**a**) chitosan, Mg_3_Al and chitosan/Mg_3_Al; (**b**) Mg_3_Al-IL, chitosan/Mg_3_Al-IL and chitosan/Mg_3_Al/IL.

The thermoanalytical curves of the synthesized hybrid materials compared to the raw materials (chitosan, Mg_3_Al, Mg_3_Al-IL) are presented in Figure 4.

For chitosan, on the TGA curve, three stages of the mass loss process are observed. The first stage occurs between 20 and 150 °C with a mass loss of 9.88%. This mass loss is due to the water adsorbed by chitosan, and an endothermic peak is noted on the DSC diagram with a maximum at 81 °C. The second stage of mass loss, between 200 and 400 °C, is due to the partial decomposition of chitosan, with a mass loss of 46.7%. Since the products resulting from decomposition are volatile, the strong exothermic effect on the DSC curve, with a maximum at 305 °C, is attributed to the combustion of these compounds. The third stage of mass loss, amounting to 41.9%, occurs between 400 and 600 °C and is due to the complete decomposition of chitosan and the pyrolysis of the decomposition products. An exothermic peak is observed on the DSC diagram with a maximum at 567 °C. Literature studies indicate that the pyrolysis process of chitosan begins with the cleavage of glycosidic bonds, followed by the formation of acetic and butyric acids and small-molecule fatty acids [37]. The total mass loss is 98.5%; practically, chitosan completely decomposes by 600 °C.

The Mg_3_Al layered double hydroxide presents two stages of mass loss on the TGA curve. In the first stage, occurring between 20 and 250 °C, the mass loss is 17.7% and is attributed to the loss of water adsorbed on the surface of the material and crystallization water (present in the interlayer space). On the DSC curve, this mass loss is accompanied by an endothermic effect with a peak at 188 °C. The second stage of mass loss occurs between 250 and 450 °C, and the mass lost is 28.0%. In this stage, dehydroxylation (the decomposition of hydroxides in the brucite-like layer) and decarboxylation (the decomposition of carbonate in the interlayer space) take place, and on the DSC curve, an endothermic effect is observed with a peak at 395 °C. The total mass loss is 45.7%. On the DSC diagram of Mg_3_Al, two more endothermic shoulders are observed, one at 750 °C and the other at 900 °C, which are attributed to the collapse of the layered structure and the formation of mixed Mg and Al oxides (spinel) [38].

In the case of the chitosan/Mg_3_Al hybrid material, the TG curve shows three stages of the mass loss process: the first stage occurs between 20 and 225 °C, where the mass loss is 16.9%, the second stage takes place between 225 and 325 °C with a mass loss of 9.94%, and the third stage, with a mass loss of 28.5%, occurs between 325 and 450 °C. The total mass loss is 56.5%. These mass loss processes are accompanied by thermal effects on the DSC curve. Thus, between 20 and 225 °C, two endothermic peaks are observed on the DSC diagram, with maxima at 83 °C and 195 °C, attributed to the evaporation of water; the first peak is due to the evaporation of water from chitosan, while the second is due to the loss of water from the interlayer space of Mg_3_Al. The temperatures at which these thermal effects occur are higher than in the case of chitosan or Mg_3_Al, probably because hydrogen bonds are formed between the two different structures in the chitosan/Mg_3_Al composite material. In the temperature range of 225–325 °C, an exothermic shoulder is observed at 220 °C, followed by a strong exothermic peak at 268 °C and an exothermic shoulder at 304 °C, all due to the decomposition of chitosan and the combustion of pyrolysis products. The presence of the layered double hydroxide in the composite structure caused the combustion process of the chitosan decomposition products to occur at a lower temperature and gradually, in three steps, rather than in a single stage as in chitosan (see Figure 4a). In the temperature range of 325–450 °C, an endothermic peak is observed at 404 °C, attributed to the dehydroxylation and decarbonation of Mg_3_Al, as well as an exothermic shoulder at 440 °C due to the complete decomposition of chitosan. The decomposition temperature of the layered double hydroxide is higher in the chitosan/Mg_3_Al composite than in Mg_3_Al, while the decomposition temperature of chitosan is lower in the chitosan/Mg_3_Al composite than in chitosan, likely due to the interaction between the two components of the composite.

On the TGA curve, Mg_3_Al-IL exhibits two stages of mass loss: the first stage occurs between 20 and 200 °C, with a mass loss of 12.5%, and the second stage occurs between 200 and 550 °C, with a mass loss of 35.3%. The total mass loss is 47.9%. The thermal effects occurring during the heating of the sample are visible on the DSC curve of the Mg_3_Al-IL material. In the range of 20–200 °C, an endothermic peak is observed at 129 °C, attributed to the loss of water from the structure of the layered double hydroxide. In the range of 200–500 °C, a succession of decomposition processes reflects the decomposition of the ionic liquid and the layered double hydroxide. Specifically, at 200 °C, an exothermic shoulder is observed, followed by an exothermic peak at 256 °C and another exothermic shoulder at 300 °C, all due to the decomposition of the ionic liquid and the combustion of decomposition products. At 360 °C, an endothermic shoulder is noted, followed by an endothermic peak at 393 °C and another endothermic shoulder at 480 °C, all attributed to the decomposition of the layered double hydroxide. Compared to the Mg_3_Al material, which shows a single endothermic peak at 395 °C due to both dehydroxylation and decarbonation (see Figure 4b), the presence of three endothermic effects in Mg_3_Al-IL indicates that the dehydroxylation and decarboxylation processes occur successively, likely due to the ionic liquid present between the layers of the layered double hydroxide. At approximately 700 °C, an endothermic shoulder is observed, attributed to the collapse of the layered structure of the layered double hydroxide.

On the TGA curve for chitosan/Mg_3_Al-IL, four temperature ranges are observed where mass loss occurs: 20–200 °C: mass loss of 6.35%; 120–240 °C: mass loss of 10.1%; 240–300 °C: mass loss of 9.85%; 300–600 °C: mass loss of 29.9%. The total mass loss when heating this material up to 1000 °C is 56.4%. On the DSC diagram, an endothermic peak is observed at 75 °C, attributed to the loss of adsorbed water. Another endothermic peak at 206 °C is due to the loss of water from the structure of the layered double hydroxide. A strong exothermic peak at 263 °C and an exothermic shoulder at 320 °C are attributed to the decomposition of both chitosan and the ionic liquid, accompanied by the combustion of decomposition products. The endothermic peak at 408 °C is due to the decomposition (dehydroxylation and decarboxylation) of the layered double hydroxide. Additionally, an exothermic shoulder at 520 °C corresponds to the complete decomposition of chitosan, while an endothermic shoulder at 700 °C is attributed to the collapse of the layers of the layered double hydroxide and the formation of mixed oxides of Mg and Al.

The thermogravimetric analysis of the chitosan/Mg_3_Al/IL material reveals that, upon heating the material to 1000 °C in air, four mass loss processes occur. The first mass loss process is between 20 and 120 °C, with a mass loss of 17.2%. This process is due to the loss of adsorbed water and is accompanied by an endothermic effect on the DSC diagram, with the endothermic peak reaching its maximum at 76 °C. The second mass loss process occurs between 120 and 230 °C, with a mass loss of 9.05%, and is attributed to the loss of water from the interlayer space of the layered double hydroxide. This process is accompanied by an endothermic peak with a maximum at 192 °C. Between 230 and 300 °C, the third mass loss process takes place, with a mass loss of 11.5%. A strong exothermic process at 270 °C accompanies this mass loss and is due to the decomposition of chitosan and the ionic liquid, processes that occur simultaneously with the combustion of the decomposition products. The fourth mass loss process occurs between 300 and 600 °C, with a mass loss of 20.6%, and is accompanied by an exothermic shoulder at 350 °C due to the pyrolysis of chitosan, an endothermic peak at 405 °C due to the dehydroxylation and decarbonation of the layered double hydroxide, and an exothermic shoulder at 520 °C due to the complete decomposition of chitosan and the pyrolysis of the decomposition products. Additionally, on the DSC curve, a shoulder at 700 °C is observed, attributed to the collapse of the layered double hydroxide structure and the formation of mixed Mg and Al oxides. The total mass loss of this material is 58.4%. The maximum temperatures at which the endothermic and exothermic effects occur in the chitosan/Mg_3_Al/IL material differ from those observed in the chitosan/Mg_3_Al-IL material, likely due to the different bonding between chitosan, the layered double hydroxide, and the ionic liquid, as the materials were synthesized differently. The results show great correlation with the conclusions raised from the FTIR analysis.

### 2.3. Kinetic Studies on Pd Adsorption on the Studied Materials

**Figure 5:**
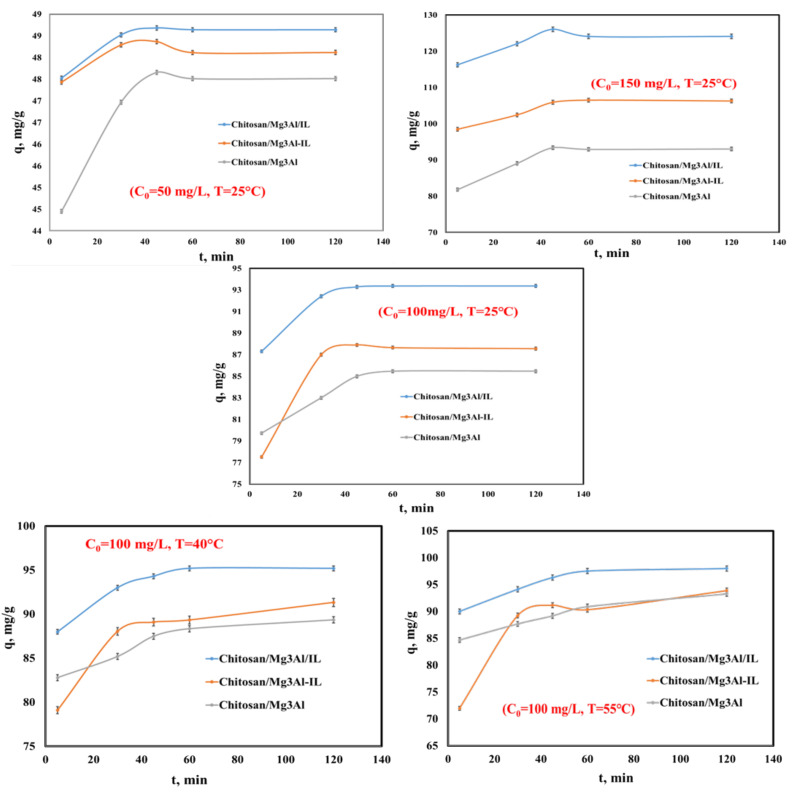
The dependence of the adsorption capacity of the studied materials versus stirring time, using 3 different initial concentrations of Pd solutions (C_0_ = 50 mg/L, C_0_ = 100 mg/L, C_0_ = 150 mg/L) at T = 25 °C and for C_0_ = 100 mg/L at three temperatures (T = 25 °C, 40 °C and 55 °C).

Exploring how the duration of the adsorption process affects adsorption capacity is significant as it offers insights into when an adsorption equilibrium is reached. Additionally, these studies help in identifying the variables that impact the adsorption of the target compound, in this case palladium. The experimental data regarding the dependence of the adsorption capacity of the studied materials according to the contact time for three initial concentrations of palladium at the temperature of 25 °C, and for the initial concentration of 100 mg/L at three temperatures are presented in Figure 5.

The results indicate a clear relationship between contact time and adsorption capacity for the studied materials. The adsorption capacity of the materials rises as the contact time increases up to 60 min for all studied materials, initial palladium concentrations and temperatures. Beyond this point, the capacity stabilizes at higher time values. This trend could be attributed to the gradual occupation of active sites on the surface of the adsorbents. Initially, many active sites are available for palladium ions to bind to, leading to a rapid increase in adsorption capacity [39]. However, as more sites become occupied, the rate of adsorption slows, resulting in stabilization of capacity at longer time intervals. At higher temperatures, a slowly increased adsorption capacity was achieved for all the hybrid materials using an initial concentration of 100 mg/L Pd. Higher temperatures typically increase the kinetic energy of the palladium ions in the solution. This enhanced kinetic energy leads to more frequent and vigorous collisions between the palladium ions and the active sites on the surface of the adsorbents. As a result, the likelihood of successful adsorption events increases, contributing to the observed rise in capacity.

The kinetics of adsorption plays a pivotal role in determining the effectiveness of the adsorption procedure. To understand how palladium is adsorbed onto the three materials being studied and determine the most suitable model that aligns with the experimental results, three kinetic models were employed. These models include the pseudo-first-order kinetic model, the pseudo-second-order kinetic model, and the intraparticle diffusion model known as Weber–Morris [35,40].

**Figure 6:**
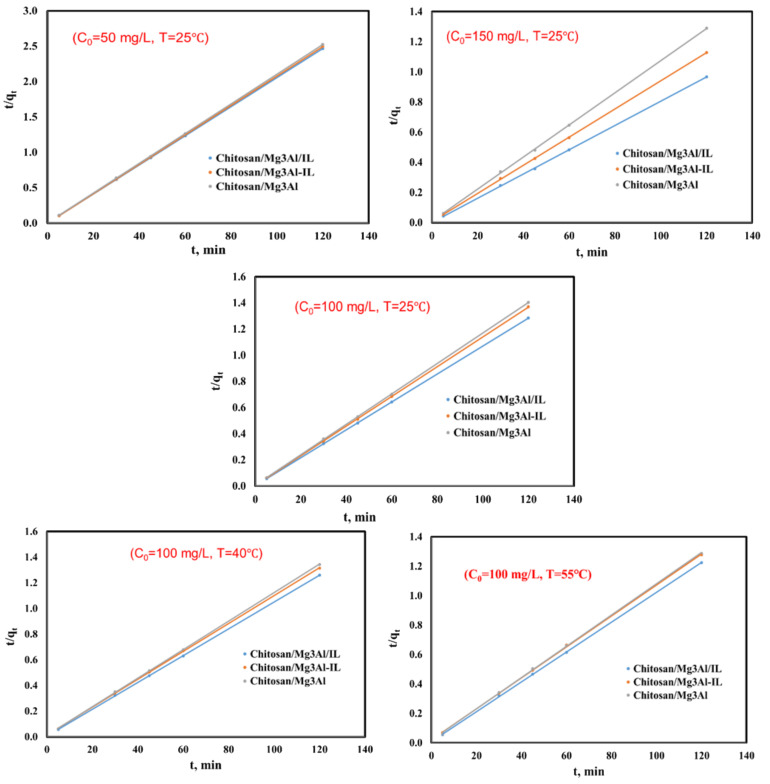
Linear representation of the pseudo-second-order kinetic model for palladium adsorption onto the studied materials, using 3 different initial concentrations of Pd solutions (C_0_ = 50 mg/L, C_0_ = 100 mg/L, C_0_ = 150 mg/L) at T = 25 °C and for C_0_ = 100 mg/L at three temperatures (T = 25 °C, 40 °C and 55 °C).

**Figure 7:**
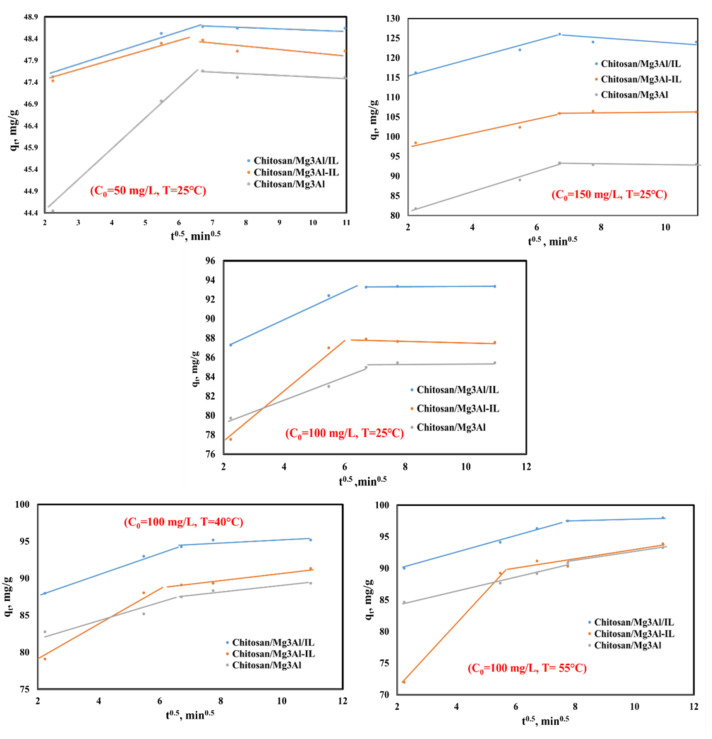
Representation of the intraparticle diffusion kinetic model for palladium adsorption onto the studied materials using 3 different initial concentrations of Pd solutions (C_0_ = 50 mg/L, C_0_ = 100 mg/L, C_0_ = 150 mg/L) at T = 25 °C and for C_0_ = 100 mg/L at three temperatures (T = 25 °C, 40 °C and 55 °C).

From the linear dependencies ln(q_e_ − q_t_) as a function of t, rate constants k_1_ were calculated (figures omitted for the sake of brevity). The graphical representations of t/q_t_ as a function of time t allowed the evaluation of the rate constants k_2_ (from the slope of the lines) of the amount of palladium adsorbed at equilibrium q_e_ (from the ordinate to the origin) (Figure 6). The values of the rate constants k_int_ were determined from the slopes of the representation’s q_t_ as a function of t^1/2^ (Figure 7). The experimental values and calculated results of the adsorption capacity at equilibrium, q_e_, the values of the rate constants and the regression coefficients for all the studied cases are presented in Table 1.

The data presented in Table 1 indicate that the application of the pseudo-first-order kinetic model resulted in significant differences between the experimentally determined equilibrium adsorption capacity values and those predicted by the model. This inconsistency is particularly evident when examining the regression coefficients, which serve as indicators of how well the model fits the experimental data. In a pseudo-first-order kinetic model, it is assumed that the rate of adsorption is proportional to the number of unoccupied sites on the adsorbent surface. This model typically applies well to systems where the adsorption process is rapid and dominated by surface interactions. However, the considerable differences observed between the experimental and modeled values suggest that this assumption may not hold true for the palladium adsorption process on the materials studied. The lower regression coefficients further reinforce this conclusion. In light of these findings, it is clear that the pseudo-first-order kinetic model is not suitable for accurately describing the adsorption kinetics of palladium on the studied materials. This suggests that alternative kinetic models, such as the pseudo-second-order model or intraparticle diffusion model, may provide a better fit and a more accurate representation of the adsorption process.

The analysis of the linear dependencies of t/qt as a function of time, as illustrated in Figure 6, indicates a good fit between the pseudo-second-order kinetic model and the experimental data. The correlation coefficients derived from these linear representations are notably close to 1 across all experimental conditions studied. Furthermore, the adsorption capacity values obtained experimentally are in close proximity to those predicted by the pseudo-second-order model. This congruence reinforces the validity of the model, as it suggests that the pseudo-second-order kinetics effectively reflect the actual behavior of the system. The close proximity of experimental and modeled values indicates that the model can reliably predict the amount of palladium that can be adsorbed under various conditions. This finding highlights that the adsorption mechanism may involve significant interactions between the palladium ions and the adsorbent surfaces, potentially including chemisorption processes [31,41]. The highest value for the constant of the pseudo-second order kinetic model, K_2,_ was obtained in the case of chitosan/Mg_3_Al/IL, suggesting an increased rate of adsorption; in this case, the palladium ions are being rapidly taken up by the adsorbent. This finding suggests an enhanced affinity between the adsorbent material and the palladium ions, reflecting highly efficient interactions at the active sites. Notably, the synthesis method employed—incorporating all three components simultaneously—results in the formation of adsorbent materials with a greater number of active sites. This configuration not only contributes to an increased adsorption capacity but also fosters more robust interactions and a higher rate of adsorption.

The analysis of the relationship between q_t_ and t^1/2^ (the square root of time), as depicted in Figure 7, reveals that the palladium adsorption process on the studied materials operates through a complex mechanism characterized by two distinct stages. This dual-stage behavior is evidenced by the presence of two slopes in the plotted diagram, indicating different phases of the adsorption process.

The first stage, represented by the steeper slope, corresponds to an instantaneous adsorption process. This phase is primarily governed by the rapid diffusion of palladium ions toward the external surface of the adsorbent materials. During this initial phase, the available active sites on the surface are quickly occupied by the solute, leading to a swift increase in adsorption capacity. This rapid interaction suggests that external mass transfer is a significant factor in the early stages of adsorption, where the concentration gradient drives the movement of palladium ions towards the adsorbent surface. In contrast, the second stage is characterized by a shallower slope, indicating a slower rate of adsorption. This phase can be attributed to the gradual adsorption that occurs within the porous structure of the adsorbent materials. As the external sites become saturated, the process transitions to a more complex mechanism involving intra-particle diffusion, where palladium ions must navigate through the pores of the adsorbent to reach deeper active sites. This phase is often considered the rate-determining step of the adsorption process, as it involves overcoming additional barriers, such as pore diffusion resistance and potential interactions with the adsorbent matrix. Furthermore, the values of the C parameter suggest the presence of a thicker boundary layer surrounding the adsorbent particles. Notably, this value is elevated in the case of hybrid materials incorporating ionic liquids, indicating that these materials are enveloped by a greater density of active functional groups. This abundance of active sites may lead to a longer diffusion path for the adsorbate, as the initial interactions involve the formation of chemical bonds between the palladium ions and the functional groups on the adsorbent. The identification of these two distinct stages highlights the multifaceted nature of the adsorption process for palladium on the studied materials. It suggests that both external diffusion and internal pore dynamics play crucial roles in determining the overall efficiency and capacity of the adsorption system [35].

In summary, the combined insights from the pseudo-second-order kinetic model and the two-stage adsorption mechanism elucidate the multifaceted nature of palladium adsorption on the studied materials. The findings suggest that effective adsorption not only involves rapid surface interactions but also requires consideration of the complexities associated with pore diffusion.

### 2.4. Thermodynamic Studies on Pd Adsorption on the Studied Materials

**Figure 8:**
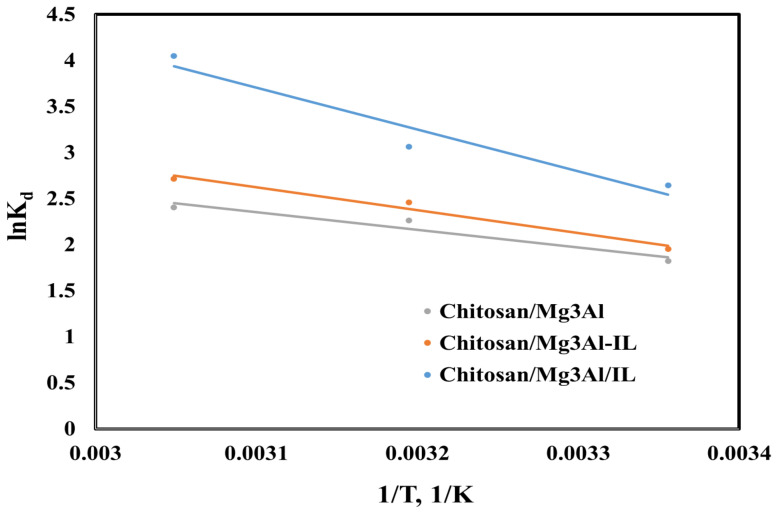
Van’t Hoff’s linearized equation representation of palladium adsorption onto the studied hybrid materials.

**Figure 9:**
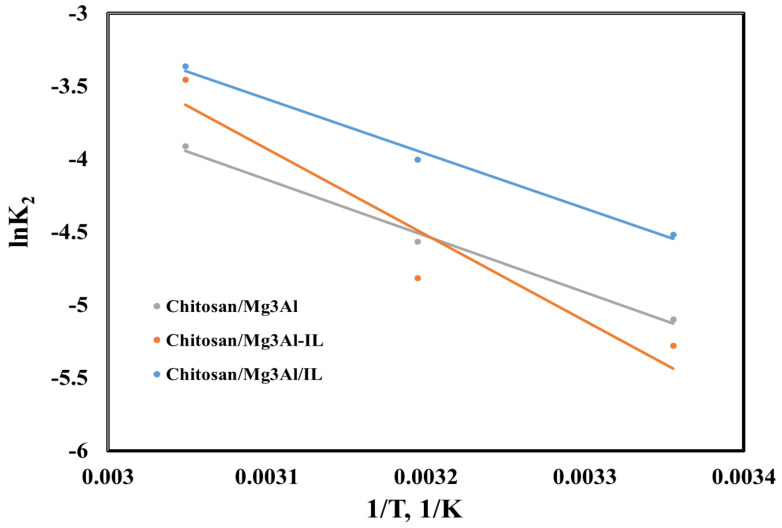
Representation of the linearized Arrhenius equation for palladium adsorption onto the studied hybrid materials.

To study the nature of the palladium adsorption process on the studied materials, the thermodynamic parameters (enthalpy, entropy and Gibbs free energy) were calculated [26]. By analyzing the relationship between the equilibrium constant (K_d_ = q_e_/C_e_) and temperature, and by representing the Van’t Hoff linear plot (Figure 8), the slope allows us to calculate the enthalpy change ΔH^0^, and the intercept can be used to determine the entropy change ΔS^0^. The activation energy (Ea) was calculated using the linearized form of the Arrhenius equation (Figure 9) [28].The obtained results are presented in Table 2.

In all cases, ΔG^0^ is negative, indicating that the reactions are spontaneous. The positive values of the entropy suggest that the system transitions from a more ordered state to a more disordered one as the adsorption progresses, reflecting an increase in randomness or functional site freedom. This increase in disorder is often associated with the breaking of structured interactions. The enthalpy values are positive, which means that the reactions are endothermic. Furthermore, the observed activation energy values exceeding 4.2 kJ/mol imply that the adsorption of palladium (Pd) onto the studied materials is characterized by chemisorption, a process where strong chemical bonds are formed between the adsorbate and the surface. This suggests that the interaction is not merely physical but involves significant rearrangement of electrons and bonds, typically resulting in more stable and specific interactions. For adsorbent materials containing ionic liquids, the activation energy is notably higher compared to hybrid materials lacking ionic liquids. This finding strongly indicates that the chemical interactions are significantly more pronounced in adsorbent materials incorporating ionic liquids. This observation suggests that the presence of ionic liquids not only intensifies the strength of these interactions but also increases their frequency, thereby facilitating and enhancing the overall efficacy of the adsorption process. Overall, these thermodynamic and kinetic parameters provide a comprehensive understanding of the adsorption behavior of Pd on the studied materials, highlighting the complex interplay between spontaneity, entropy, enthalpy, and the nature of the adsorption process [28].

### 2.5. Equilibrium Studies on Pd Adsorption on the Studied Materials

**Figure 10:**
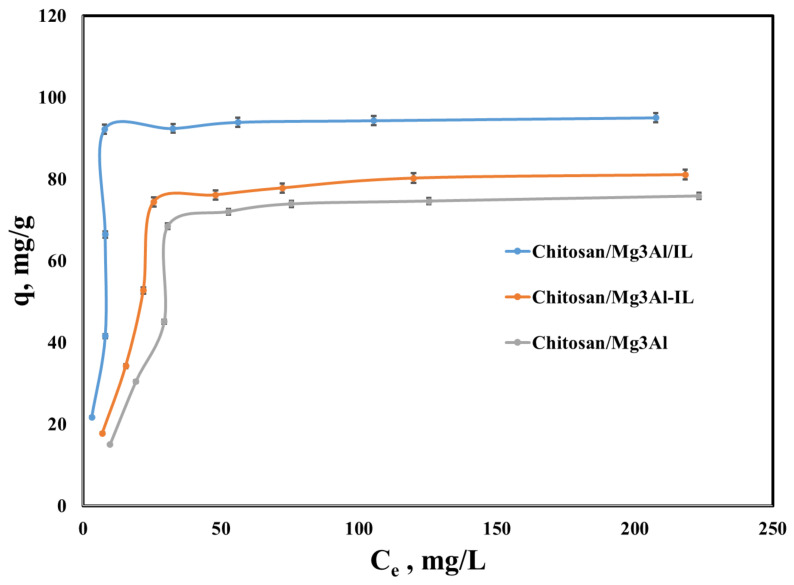
Equilibrium isotherms for palladium adsorption onto the studied hybrid materials.

Equilibrium studies convey information on the maximum adsorption capacity and affinity of the materials used as adsorbents with respect to the target compound. Figure 10 presents the equilibrium isotherms for palladium adsorption onto the studied hybrid materials. The adsorption capacity developed by the studied materials increases with rising equilibrium concentration until reaching a constant value, which corresponds to the saturation of the adsorbent surface with palladium ions. Notably, the lowest adsorption capacity was observed for chitosan/Mg_3_Al at an initial concentration of 200 mg/L. The functionalization of the adsorbents with the studied ionic liquid significantly enhances their adsorptive efficiency, indicating that the functional groups in the ionic liquid play a key role in facilitating the removal of palladium from aqueous solutions. Furthermore, the method of preparing the adsorbent material was found to impact the effectiveness of palladium recovery. Among the materials tested, the chitosan/Mg_3_Al/IL hybrid exhibited the highest adsorption capacity, suggesting that the ionic liquid functionalization is most beneficial when applied during the hybrid material synthesis, rather than during the LDH synthesis process.

**Figure 11:**
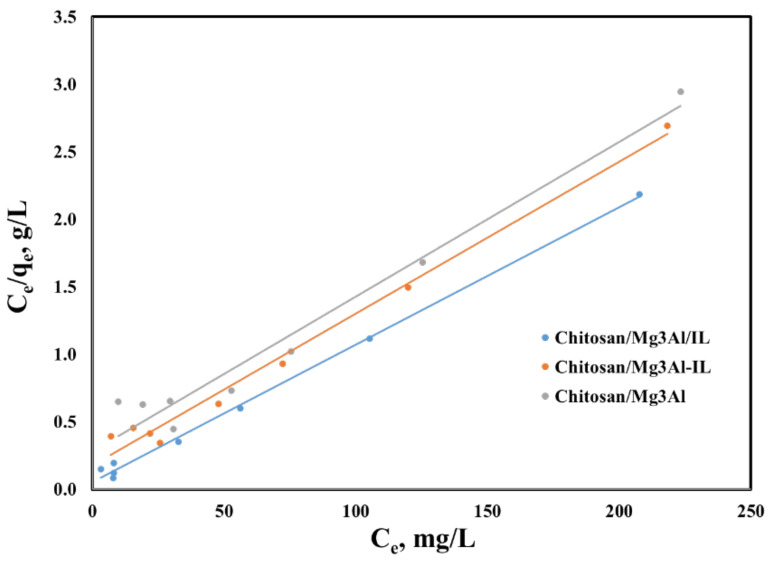
Langmuir isotherm for palladium adsorption onto the studied hybrid materials.

The modelling of experimental isotherms helps to identify the mechanism of the process and allows the evaluation, theoretically, of the adsorption capacity at equilibrium. For this purpose, Langmuir and Freundlich isotherms were used in the presented studies [36]. The Langmuir isotherm describes an adsorption process in a monomolecular layer on a surface containing a finite number of energetically equivalent active centers. It is best suited for systems where adsorption occurs at specific sites with no interaction between adsorbed molecules. The Langmuir isotherm considers the effect of surface saturation, assimilating adsorption with a chemical phenomenon. The q_m_ and K_L_ values are determined from the graphic representation of C_e_/q_e_ as a function of C_e_ (Figure 11). The Freundlich isotherm is the empirical expression that describes the adsorption equilibrium on energetically inhomogeneous surfaces, being valid for systems where the adsorption sites have varying affinities for the adsorbate. This model is typically applied to adsorption processes that involve multilayer adsorption, where the adsorbate molecules are adsorbed on a surface with different adsorption energies, leading to a non-uniform distribution of the adsorbate. In addition, 1/n and K_F_ are obtained from the slope and the y-intercept of the linear plot of lnq_e_ as a function of lnC_e_ (figure omitted for brevity). The parameters corresponding to the equilibrium isotherms are presented in Table 3, along with the R_2_ correlation coefficients.

The palladium adsorption process on the studied hybrid materials is best described by the Langmuir isotherm, as evidenced by the highest correlation coefficients, which reflect the structured nature of the layered double hydroxides and the homogeneous distribution of active sites and functional groups of ionic liquid (where is the case) on their surfaces. A satisfactory alignment was observed between the experimentally determined maximum adsorption capacities and those predicted by the Langmuir model across all adsorbent materials. Conversely, the graphical representation of the experimental data as the Freundlich isotherm (figure omitted for brevity) yielded lower correlation coefficients, suggesting that the adsorption process does not conform to this one. This discrepancy is further supported by the lack of agreement between the experimentally obtained maximum adsorption capacities and the calculated values.

Notably, the 1/n parameter of the Freundlich isotherm, which is consistently below one for all materials studied, indicates a strong affinity of these adsorbents for palladium. Additionally, the maximum adsorption capacities of the materials increase in the following order: chitosan/Mg_3_Al < chitosan/Mg_3_Al-IL < chitosan/Mg_3_Al/IL.

Different values of the maximum adsorption capacity of palladium ions, determined from the Langmuir isotherm, using other adsorbent materials, are presented in Table 4.

Existing literature underscores the significant advantages of employing ionic liquids for the functionalization of various materials, which enhances the presence of functional groups on the surfaces of adsorbents, thereby improving their adsorption efficiency. The results obtained from the studied hybrid materials demonstrate comparable, if not superior, performance in palladium ion recovery when compared with other functionalized materials documented in the literature. Notably, hybrid materials exhibit enhanced efficiency in recovering palladium ions from aqueous solutions, even at low concentrations. This enhanced performance can be attributed to the unique structural characteristics of the hybrid materials, which facilitate the interactions between the adsorbent and adsorbate and enhance strength and resistance during the adsorption process. These hybrid materials incorporating chitosan have been meticulously developed to facilitate the recovery of palladium from aqueous solutions through column studies. While materials composed solely of layered double hydroxides (LDHs) and ionic liquids have been previously employed [23,24], and sometimes with higher adsorption capacity for Pd removal, their fine structure presents significant challenges for future column applications, as it can lead to column clogging. By integrating chitosan, the aim is to enhance the mechanical properties of the adsorbent, thereby ensuring its suitability for effective use in column studies. Looking ahead, our future goals include scaling up the palladium recovery process from laboratory to micro pilot scale, utilizing continuous adsorption techniques. Additionally, we aspire to repurpose the recovered palladium from low-concentration solutions, transforming the resultant materials into valuable photocatalytic agents. Consequently, these materials hold promise for future applications in continuous treatment systems, offering a distinct advantage over presented powdered adsorbents, as highlighted in Table 4. This capability not only enhances operational efficiency but also contributes to sustainable resource management in the recovery of precious metals.

## 3. Materials and Methods

### 3.1. Synthesis of Adsorbent Materials

To synthesize the layered double hydroxide, Mg_3_Al, the co-precipitation method at a low supersaturation was employed [44,45]. Mg(NO_3_)_2_·6H_2_O and Al(NO_3_)_3_·9H_2_O (Merck—Milipore, Darmstadt, Germany) served as a metal ion source, maintaining a Mg^2+^/Al^3+^ ratio of 3:1. A 200 mL nitrate mixture was prepared in the correct stoichiometric ratio. This mixture was added dropwise under magnetic stirring to a 1M Na_2_CO_3_ solution in a Berzelius beaker. The precipitation pH, crucial for the process, was kept between 9.5 and 10.5 by adding a 2 M NaOH solution dropwise. After adding all the metals and achieving the desired pH, the reaction mixture was matured in an oven at 70 °C for 24 h. The precipitate was then filtered and washed with distilled water until a neutral pH was reached. The samples were dried in the oven under the same conditions, then mortared and sieved to use only the <90 µm fraction.

For the functionalized compound with 10% ionic liquid (methyl trialchil ammonium chloride) (Sigma-Aldrich Chemie GmbH—München, Germany), the same method was used, but the nitrate mixture was added dropwise to an ionic liquid solution dissolved in acetone (ionic liquid:acetone = 1 g/50 mL). The pH was maintained using a NaOH solution prepared with boiled and cooled water to prevent carbonate ions from entering the LDH interlayer space and to allow ionic liquid molecules access.

To enhance the mechanical strength of the adsorbents (Mg_3_Al and Mg_3_Al-IL), they were dispersed in a chitosan solution dissolved in 1% acetic acid to create the following hybrid materials: chitosan/Mg_3_Al, chitosan/Mg_3_Al-IL, and chitosan/Mg_3_Al/IL. The chitosan:LDH ratio was 1:4. A 500 mL 1% CH_3_COOH solution was prepared, and 1 g of chitosan was added under stirring until fully dissolved (about 15 min). Then, 4 g of Mg_3_Al or Mg_3_Al-IL was added. Due to the acidic pH, the adsorbents dissolved and then were reprecipitated by neutralizing the solution with 1M NaOH, stirring for 2 h. The alkaline solution was added dropwise to the chitosan/Mg_3_Al or chitosan/Mg_3_Al-IL mixture until a pH of 9.8–10.2 was achieved. The precipitate was washed and dried. A hybrid material of chitosan, Mg_3_Al, and IL was also prepared using the same method, with the ionic liquid dissolved in acetone added to the chitosan acid solution. Table 5 provides an overview of the symbols and techniques utilized to acquire hybrid materials under investigation.

### 3.2. Characterization of the Obtained Hybrid Materials

The investigated hybrid materials were characterized using X-ray diffraction, Fourier transform IR spectroscopy (FTIR), scanning electron microscopy (SEM) with an energy dispersive X-ray detector (EDX) and thermal analysis. X-ray powder diffraction patterns were recorded using a Rigaku (Tokyo, Japan) Ultima IV diffractometer (40 kV, 40 mA) with CuKα radiation at a scan rate of 5°/min and a step size of 0.01 in the range 2θ = 2–80°. FTIR spectra were recorded using a Shimadzu (Kyoto, Japan) Prestige-21 spectrophotometer in the wavenumber range 4000–400 cm^−1^. SEM images were taken using a Quanta FEG 250 microscope (FEI, Hillsboro, OR, USA) equipped with an EDAX/ZAF quantifier (EDAX, Pleasanton, CA, USA). The thermal analysis curves of the synthesized materials were recorded using NETZSCH STA 449 C equipment (NETZSCH, Selb, Germany), in an air atmosphere, at a rate of 5°/min and in the temperature range of 20–1000 °C.

### 3.3. Palladium Recovery Studies

Research on palladium ion adsorption from aqueous solutions using the synthesized hybrid materials was conducted in a discontinuous process at 200 rpm using a Julabo (Seelbach, Germany) SW23 stirring bath at 25 °C. Palladium is present in solution as Pd^2+^, which presents a low solubility at pH higher than 5. Therefore, to avoid its removal from solution through precipitation, it is important to work at pH values lower than 5. By employing literature research, it was observed that the optimum pH of Pd adsorption is Ph = 3–4, regardless of the nature of the adsorbent materials. At pH lower than 3, the structure of the layered double hydroxide collapsed. Considering these limits, we chose to work at an initial pH = 3.5 for the Pd solutions [20,21,26,27,28]. The pH of the solutions was adjusted with a 2M NaOH and HCl solution, in a dropwise manner, thereby minimizing the potential competition between Na and H ions during the adsorption process of Pd. The effectiveness of the process was calculated based on the adsorption capacity of the materials relative to contact time, temperature, and initial palladium ion concentration (Pd(II)). Adsorption capacities were calculated using the following equation [40]:

$$q_{t}=\frac{(C_{0}-C_{t})\cdot V}{m}$$

where *C*_0_—initial concentration of palladium, in solution (mg/L);

*C_t_*—the palladium concentration in the solution, after a time t (mg/L);

*V*—the volume of the solution (L);

*M*—mass of the adsorbent used (g).

Equilibrium time was determined using a solid/liquid ratio of 1 g/L and Pd(II) solutions with concentrations of 50, 100, and 150 mg/L at 25 °C. Samples were kept in contact for different times (5, 30, 45, 60, and 120 min), filtered, and the residual Pd concentration was measured by atomic absorption spectrometry using a Varian SpectrAA 280 FS spectrometer (Agilent Technologies, Santa Clara, CA, USA).

The kinetic studies were discussed using the pseudo-first-order, pseudo-second-order and intra particle diffusion kinetic models.

The integrated form of the Lagergren model was used, described by the following relation [46,47]:

$$\ln\left(q_{e}-q_{t}\right)=\ln q_{t}-k_{1}\cdot t$$

where *q_t_*—adsorption capacity of the adsorbent at time t, mg/g;

*q_e_*—adsorption capacity of the adsorbent at equilibrium, mg/g;

*k*_1—_adsorption speed constant, min^−1^;

*t*—time, min.

The pseudo-second-order kinetic model, proposed by Ho and Mckay, is expressed by the following equation [48]:

$$\frac{t}{q_{t}}=\frac{1}{k_{2}\cdot q_{e}^{2}}+\frac{t}{q_{e}}$$

where *k*_2_—the rate constant of the pseudo-second-order adsorption kinetic model (g/mg∙min);

*q_e_*—the amount of palladium adsorbed at equilibrium (mg/g);

*q_t_*—the amount of palladium adsorbed at time t (mg/g).

The kinetic model of intraparticle diffusion is described by the Weber–Morris equation that was used to identify the rate-determining step where intraparticle diffusion describes the kinetics of the process [49].

$$q_{t}=K_{int}\cdot t^{\frac{1}{2}}+C$$

where *k*_int_ is the rate constant of the kinetic model of intraparticle diffusion (mg/g∙min^1/2^).

Thermodynamic studies used a 100 mg/L Pd solution, varying the temperature (25, 40, and 55 °C) while keeping the other parameters constant (S/L ratio, stirring time, speed, pH).

The thermodynamic parameters (enthalpy, entropy and Gibbs free energy) were calculated using the following equations [49]:

$$\Delta G^{0}=\Delta H^{0}-T\Delta S^{0}$$

$$\;lnK_{d}=\frac{\Delta S^{0}}{R}-\frac{\Delta H^{0}\;}{RT}$$

$$\;\;\;\;\;\;\;K_{d}=\frac{q_{e}}{C_{e}}$$

where Δ*G*^0^—Gibbs free energy (KJ/mol);

Δ*S*^0^—entropy (J/(molK));

Δ*H*^0^—enthalpy (KJ/mol);

*T*—absolute temperature (K);

*R*—universal gas constant (8.314 J/(mol∙K));

*K_d_*—distribution coefficient.

The activation energy (E_a_, J/mol) was calculated using the linearized form of the Arrhenius equation, as shown below [50]:

$$\;\;lnk_{2}=lnA-\frac{E_{a}}{RT}\;$$

where A—constant for each chemical reaction, which refers to the frequency with which collisions with optimal orientation occur.

Equilibrium studies were conducted with different initial Pd(II) concentrations (25, 50, 75, 100, 125, 150, 200, and 300 mg/L). The experimental data were fitted with Langmuir and Freundlich isotherms.

The linearized form of the Langmuir isotherm corresponds to the following equation [50]:

$$\frac{C_{e}}{q_{e}}=\frac{1}{q_{m}K_{L}}+\frac{C_{e}}{q_{m}}$$

where *q_e_*—the amount of palladium retained at equilibrium, mg/g;

*q_m_*—the maximum amount of adsorbate retained for full coverage at the level of monolayer, mg/g;

*C_e_*—concentration at equilibrium, mg/L;

*K_L_*—the Langmuir constant (adsorption coefficient) related to the adsorption energy, L/mg.

The linear form of the Freundlich isotherm is given by the following equation [51]:

$$\ln q_{e}=\ln K_{F}+\frac{1}{n}\ln C_{e}$$

where *q_e_*—adsorption capacity at equilibrium, mg/g;

*C_e_*—the concentration of the adsorbed in the solution, at equilibrium, mg/L;

*K_F_*—Freundlich constant, L/mg;

*ln*—inhomogeneity factor (related to the adsorption energy), dimensionless. All adsorption experiments were conducted in triplicate to ensure accuracy and reliability of the results.

## 4. Conclusions

This study has demonstrated the remarkable potential of hybrid materials composed of layered double hydroxides (LDHs), chitosan, and ionic liquids for the efficient adsorption of palladium ions from low-concentration aqueous solutions. The comprehensive characterization of these materials revealed their structural integrity and functional capabilities, which contributes to their enhanced performance in palladium recovery. Kinetic and thermodynamic analyses provided insights into the adsorption mechanisms, confirming that the process is predominantly governed by chemical sorption involving the multifaceted nature (external diffusion and internal pore dynamics) of the adsorption process for palladium onto the studied materials. Notably, the equilibrium studies indicated that the adsorption of palladium onto the hybrid materials is best described by the Langmuir isotherm model, suggesting a homogeneously distributed surface with finite active sites.

Importantly, the results obtained from the hybrid materials exhibited comparable, even superior, efficiency in palladium recovery when juxtaposed with other functionalized materials reported in the literature. This efficiency is particularly pronounced in the context of low-concentration aqueous solutions, underscoring the practical applicability of these materials in real-world scenarios. Furthermore, the unique structural properties of the hybrid materials enhance their resistance and stability, paving the way for their further potential use in continuous treatment systems, a significant advancement over listed powdered adsorbents. The promising results advocate for further exploration and development of these materials for industrial applications, ultimately supporting the recovery of precious metals and the advancement of green chemistry practices.

## Figures and Tables

**Table 1:** The kinetic parameters resulting from the application of the kinetic models regarding palladium adsorption on the studied materials, using 3 different initial concentrations of Pd solutions (C_0_ = 50 mg/L, C_0_ = 100 mg/L, C_0_ = 150 mg/L) at T = 25 °C and for C_0_ = 100 mg/L at three temperatures (T = 25 °C, 40 °C and 55 °C).

C_0_ = 50 mg Pd/L, T = 25 °C										
Adsorbant	q_e_ exp.	Pseudo Order I			Pseudo Order II			Intraparticle Diffusion		
Materials	mg/g	q_e_ calc.	K_1_	R^2^	q_e_ calc.	K_2_	R^2^	K_int_, mg/g	C	R^2^
		mg/g	min^−1^		mg/g	min/(mg/g)		min^−1/2^		
Chitosan/Mg_3_Al/IL	48.6	0.32	0.0209	0.5404	48.7	0.2626	0.9998	0.2685	46.9	0.9802
Chitosan/Mg_3_Al-IL	48.3	0.22	0.0029	0.7294	48	0.3605	0.9978	0.221	46.9	0.9622
Chitosan/Mg_3_Al	47.5	0.953	0.0207	0.699	47.6	0.0773	0.9996	0.7293	42.8	0.996
C_0_ = 100 mg Pd/L, T = 25 °C										
	q_e_ exp.	q_e_ calc.	K_1_	R^2^	q_e_ calc.	K_2_	R^2^	K_int_, mg/g	C	R^2^
	mg/g	mg/g	min^−1^		mg/g	min/(mg/g)		min^−1/2^		
Chitosan/Mg_3_Al/IL	93.2	2.38	0.0423	0.6995	93.4	0.0346	0.9999	1.3864	87.4	0.9824
Chitosan/Mg_3_Al-IL	87.5	2.1	0.0213	0.7815	87.7	0.0316	0.9997	2.4421	72.3	0.9642
Chitosan/Mg_3_Al	84.9	4.69	0.0471	0.7664	86.2	0.02	0.9995	1.1428	77	0.987
C_0_ = 150 mg Pd/L, T = 25 °C										
	q_e_ exp.	q_e_ calc.	K_1_	R^2^	q_e_ calc.	K_2_	R^2^	K_int_, mg/g	C	R^2^
	mg/g	mg/g	min^−1^		mg/g	min/(mg/g)		min^−1/2^		
Chitosan/Mg_3_Al/IL	124	0.79	0.0065	0.8826	125	0.0109	0.9999	2.1147	111	0.9576
Chitosan/Mg_3_Al-IL	105	4.51	0.03	0.8579	106	0.0051	0.9999	1.5768	94.6	0.9543
Chitosan/Mg_3_Al	93	2.76	0.0261	0.7083	93.4	0.0061	0.9999	2.5164	75.9	0.9877
C_0_ = 100 mg Pd/L, T = 40 °C										
	q_e_ exp.	q_e_ calc.	K_1_	R^2^	q_e_ calc.	K_2_	R^2^	K_int_, mg/g	C	R^2^
	mg/g	mg/g	min^−1^		mg/g	min/(mg/g)		min^−1/2^		
Chitosan/Mg_3_Al/IL	95.2	9.1	0.0854	0.7262	95.2	0.0183	0.9978	1.436	84.8	0.9947
Chitosan/Mg_3_Al-IL	91.3	33.4	0.0679	0.9279	91.7	0.0081	0.9965	2.342	74.1	0.9701
Chitosan/Mg_3_Al	89.3	38.6	0.083	0.91	90	0.0104	0.9996	0.987	80.4	0.0943
C_0_ = 100 mg Pd/L, T = 55 °C										
	q_e_ exp.	q_e_ calc.	K_1_	R^2^	q_e_ calc.	K_2_	R^2^	K_int_, mg/g	C	R^2^
	mg/g	mg/g	min^−1^		mg/g	min/(mg/g)		min^−1/2^		
Chitosan/Mg_3_Al/IL	98	9.9	0.0395	0.9717	99	0.0346	0.9995	1.3832	86.8	0.9941
Chitosan/Mg_3_Al-IL	93.8	58.6	0.0721	0.9192	95.2	0.0316	0.9995	4.5024	62.5	0.9684
Chitosan/Mg_3_Al	93.2	36.5	0.0645	0.8914	94.3	0.02	0.9996	0.9907	82.4	0.9959

**Table 2:** Thermodynamic parameters of palladium adsorption on the studied materials.

Adsorbant Materials	ΔH^0^ KJ/mol	ΔS^0^ J/mol·K	ΔG^0^, KJ/mol			R^2^	E_a_ kJ/mol	R^2^
			298 K	312 K	328 K			
Chitosan/Mg_3_Al/IL	37.8	148.15	−6.35	−8.57	−10.8	0.9348	46.5	0.9925
Chitosan/Mg_3_Al-IL	20.7	86.04	−4.94	−6.23	−7.52	0.9761	48.9	0.9099
Chitosan/Mg_3_Al	15.9	68.8	−4.601	−5.63	−6.67	0.9362	32.1	0.9926

**Table 3:** Langmuir, Freundlich, Temkin and Redlich–Peterson isotherm parameters for palladium adsorption onto the studied hybrid materials.

Isotherm		Adsorbant Materials		
		Chitosan/Mg_3_Al/IL	Chitosan/Mg_3_Al-IL	Chitosan/Mg_3_Al
q_exp_, mg/g		91.2	74.4	68.5
Langmuir	K_L_. L/mg	0.187	0.064	0.040
	q_m calc._ mg/g	98.0	88.5	85.9
	R^2^	0.9970	0.9870	0.9960
Freundlich	K_F_. mg/g	29.4	12.5	8.03
	1/n	0.26	0.41	0.48
	R^2^	0.7310	0.7030	0.7010

**Table 4:** The maximum adsorption capacities, determined from the Langmuir isotherm, present in the literature.

Adsorbant Materials	q_m calc,_ mg/g	Reference
Zn_3_Al	64.4	[27]
Zn_3_Al functionalized with methyltrialkylammonium chloride	100	[27]
MgSiO_3_ functionalized with DL-cysteine	9.23	[42]
2-Mercaptobenzothiazole functionalized Amberlite XAD-1180 resin	50.0	[41]
Silica-based adsorbent functionalized with macrocyclic ligand	83.0	[43]
Tetraoctylammonium bromide impregnated onto graphene oxide	92.7	[30]
Chitosan/Mg_3_Al/IL	98.0	The present paper
Chitosan/Mg_3_Al-IL	88.5	
Chitosan/Mg_3_Al/IL	85.9	

**Table 5:** Symbols of synthesized hybrid materials.

No.	Hybrid Material Symbol	Method
1.	Chitosan/Mg_3_Al	Co-precipitation at low supersaturation of Mg_3_Al, followed by hybrid formation by precipitating an acidic solution containing both components
2.	Chitosan/Mg_3_Al-IL	Co-precipitation at low supersaturation of Mg_3_Al-IL, followed by hybrid formation by precipitating an acidic solution containing both components
3.	Chitosan/Mg_3_Al/IL	Co-precipitation at low supersaturation of Mg_3_Al, followed by hybrid formation by precipitating an acidic solution containing all three components

## Data Availability

Data are contained within the article.
